# Gut microbiota trajectory in early life may predict development of celiac disease

**DOI:** 10.1186/s40168-018-0415-6

**Published:** 2018-02-20

**Authors:** Marta Olivares, Alan W. Walker, Amalia Capilla, Alfonso Benítez-Páez, Francesc Palau, Julian Parkhill, Gemma Castillejo, Yolanda Sanz

**Affiliations:** 1 Microbial Ecology, Nutrition and Health Research Unit, Institute of Agrochemistry and Food Technology, National Research Council (IATA-CSIC), C/Catedrático Agustín Escardino, 7. 46980, Paterna, Valencia, Spain; 2 Gut Health Group, The Rowett Institute, University of Aberdeen, Aberdeen, UK; 3 Genetics and Molecular Medicine Unit, Institute of Biomedicine of Valencia, National Research Council (IBV-CSIC), Valencia, Spain; 4 Wellcome Trust Sanger Institute, Hinxton, Cambridgeshire UK; 5 Hospital Universitari de Sant Joan de Reus, IISPV, URV, Tarragona, Spain; 6 Center for regenerative medicine, Boston university school of medicine, Boston, USA; 7 Institut de Recerca Sant Joan de Déu and CIBERER, Hospital Sant Joan de Déu, Barcelona, Spain

**Keywords:** Celiac disease, Intestinal microbiology, HLA genes

## Abstract

**Background:**

To investigate whether alterations in the developing intestinal microbiota and immune markers precede celiac disease (CD) onset in infants at familial risk of developing the disease.

**Methods:**

A nested case-control study was carried out as part of a larger prospective cohort study, which included healthy full-term newborns (> 200) with at least one first relative with biopsy-verified CD. The present study includes cases of CD (*n* = 10) and the best-matched controls (*n* = 10) who did not develop the disease after 5-year follow-up. Fecal microbiota, assessed by high-throughput 16S rRNA gene amplicon sequencing, and immune parameters were profiled at 4 and 6 months of age and related to CD onset.

**Results:**

The microbiota of infants who remained healthy showed an increase in bacterial diversity over time, characterized by increases in *Firmicutes* families, but not those who developed CD. Infants who subsequently developed CD showed a significant reduction in sIgA levels over time, while those who remained healthy showed increases in TNF-α correlated to *Bifidobacterium* spp. An increased relative abundance of *Bifidobacterium longum* was associated with control children while increased proportions of *Bifidobacterium breve* and *Enterococcus* spp. were associated with CD development.

**Conclusion:**

The findings suggest that alterations in the early trajectory of gut microbiota in infants at CD risk could influence the immune maturation process and predispose to CD, although larger population studies are warranted to confirm this hypothesis.

**Electronic supplementary material:**

The online version of this article (10.1186/s40168-018-0415-6) contains supplementary material, which is available to authorized users.

## Background

Celiac disease (CD) is an immune-mediated systemic disorder elicited by an aberrant response to dietary gluten proteins found in wheat, rye, and barley, which develops in genetically predisposed individuals [1]. The disease is strongly associated with the human leukocyte antigen *HLA-DQA1* and *HLA-DQB1* genes expressed as surface heterodimers in antigen-presenting cells (APCs). The majority of CD patients (95%) carry the HLA-DQ2.5 heterodimer, while most of the remaining patients have the HLA-DQ8 heterodimer [2]. Approximately 30–40% of the Caucasian subjects carry these risk haplotypes but most of them do not develop the disease, which has a prevalence rate of 1–3% in the general population and up to 10% in first-degree relatives of CD patients [3, 4]. Therefore, the *HLA-DQ2/DQ8* gene variants are necessary but not sufficient for the disease to develop. Although *non-HLA* variants also contribute to CD risk [4], studies on the heritability of CD in twins indicate that genetics alone cannot explain CD onset and that non-shared environmental elements also contribute [5].

Gluten intake is the only environmental trigger of CD with an established pathogenic role. APCs recognize gluten peptides and activate lamina propria infiltrating T lymphocytes. This leads to the release of pro-inflammatory cytokines, mainly interferon (IFN)γ and IL-15, activation of cytotoxic T cells, and profound tissue damage [6, 7]. Exclusion of gluten from the diet is the only treatment available, but adherence to a gluten-free diet (GFD) can be difficult. Therefore, a better understanding of modifiable factors contributing to the breakdown of gluten tolerance would be desirable to enable novel strategies for primary prevention.

The timing of gluten introduction to the infant’s diet may offer a window of opportunity for protection against CD in predisposed individuals [8, 9]; however, it has not proven effective in recent randomized intervention studies (PreventCD and CELIPREV) [10, 11]. Epidemiological data suggest that additional environmental factors, such as type of delivery at birth, milk-feeding practices, intestinal infections, and/or use of antibiotics, could also determine CD risk [12–16]. A commonality of the aforementioned factors is that they impact on gut microbiota and, thereby, may influence its role in guiding the immune system towards development, or not, of gluten tolerance [17].

Observational studies have reported alterations in the composition of the microbiota of CD patients as well as increased abundance of virulence-related genes in intestinal pathobionts [18]. Furthermore, some authors reported that some of these alterations are not due to adherence to a GFD [19] and may be associated with gastrointestinal symptoms in untreated CD [20] and GFD-treated patients [21]. The contributing role of perturbations in the gut microbiota, and of specific enteric bacteria, to gluten-induced immunopathology has been proven in animal models [22]. Further evidence from longitudinal studies in human subjects is, however, needed to elucidate the role of the gut microbiota in CD etiology. With this aim, the prospective PROFICEL study enrolled a cohort of newborn healthy infants at familial risk of developing CD to monitor the progressive assembly of gut microbiota and its temporal relationship with factors potentially contributing to CD risk. Initial studies in this cohort of infants showed that the intestinal microbiota is influenced by both milk-feeding type and the HLA-DQ genotype [23, 24].

Building upon these findings, the present study aimed to investigate whether alterations in the developing intestinal microbiota and immune markers precede CD onset in infants at familial risk of developing the disease. The ultimate goal of the study is to shed light on the interactions between modifiable environmental and heritable factors in CD with a view to helping progress towards primary prevention.

## Methods

### Study design and follow-up

A nested case-control study was carried out as part of a larger prospective cohort study (PROFICEL), which included healthy full-term newborns with at least one first relative with biopsy-verified CD. Enrollment lasted from June 2006 to November 2010, as described elsewhere [23]. The present study includes cases of CD (*n* = 10) and the best-matched controls (*n* = 10) from the cohort who did not develop the disease by the time of cases’ disease onset. Data regarding the mode of delivery, size, weight, and weeks of gestation were recorded at birth (Table 1). The health status, antibiotic intake, and feeding habits (i.e., breastfeeding and formula feeding, introduction of complementary foods) were monitored during the study period (5 years).

**Table 1:** Characteristics of the infants included in the study

	ID	Type of delivery	^a^HLA-DQ genotype	CD diagnosis (months)	Gender	Relatives with CD	Gestation (weeks)	Height (cm) at birth	Weight (g) at birth	Type of feeding at 4 months	Type of feeding at 6 months	Intake of antibiotics
Controls (*n* = 10)	C1	Vaginal	1		F	Brother	40	52	3730	FF	C + FF	
	C2	Vaginal	2		M	Brother	40	51	3750	FF	C + FF	
	C3	Vaginal	2		F	Father	40	50	3335	BF	C + BF	
	C4	Vaginal	1		F	Mother	39	48	2960	BF	C + FF + BF	
	C5	Cesarean	1		M	Mother	38	50	2800	BF	C + BF	Clarithromycin
	C6	Vaginal	2		F	Mother	39	49	2900	FF	C + FF	
	C7	Cesarean	2		M	Sister	38	51	3190	BF	C + FF + BF	
	C8	Vaginal	1		F	Father	39	49	3890	BF	C + BF	
	C9	Vaginal	2		F	Bother	38	50	3610	BF	C + BF	
	C10^d^	Vaginal	3		M	Mother	40	52	3880	BF	C + BF	
^b^Mean (SE)							39.1 (0.3)	50.2 (0.42)	3405 (133.2)			
CD cases (*n* = 10)	CD1	Vaginal	1	40	F	Mother	36	51	4890	FF	C + FF	
	CD2	Vaginal	2	30	M	Sister	40	50	3450	FF	C + FF	Augmentin
	CD3	Vaginal	2	21	M	Brother	37	48	3380	BF	C + SM	Amoxicillin
	CD4	Vaginal	1	27	F	Mother	38	48	3005	BF	C + FF	
	CD5	Cesarean	1	16	F	Sister	37	47	2730	BF	C + FF	Amoxicillin
	CD6	Vaginal	2	26	F	Mother	39	49	3200	FF	C + FF	Augmentin
	CD7	Cesarean	1	36	F	Mother	38	50	4360	BF	C + BF	
	CD8	Vaginal	1	24	M	Mother + sister	41	50	4170	BF	C + SM	Augmentin
	CD9	Vaginal	2	82	M	Mother + sister	37	48	3030	BF	C + BF	
	CD10^d^	Vaginal	2	40	F	Mother	40	47	3070	BF	C + BF	
^b^Mean (SE)				34.2 (5.9)			38.3 (0.5)	48.8 (0.44)	3529 (222.7)			
^c^*p* value		1.000	0.828	–	1.000	0.258	0.190	0.033	0.6385	1.000	0.172	0.143

*BF* breastfeeding, *FF* formula feeding, *C* complementary feeding (foods other than milk and water), *SM* soya milk

^a^Genetic risk of developing CD was established according to the HLA-DQ genotype (see the “Methods” section for details)

^b^Data of continuous variables are expressed as mean and standard error of the mean (in brackets)

^c^Statistically significant differences between cases and controls were established by applying using a *t* test for continuous variables and the chi-square test for categorical variables at *p* < 0.050

^d^No microbiota analysis available for these samples at 4 months

A total of 18 fecal samples from 9 controls and 9 CD cases were collected at 4 months, and a total number of 20 stool samples from 10 controls and 10 CD cases were collected at 6 months and analyzed in the present study. The DNA typing for CD *HLA DQA1* and *HLA DQB1* genes genotype was elucidated using sequence-specific primers (PCR-SSP) [23]. The *HLA-DQ* genotyping allowed the classification of the infants into three groups: the group 1 (high risk) included those individuals carrying the DQ2 haplotype in both *cis* (DQA1*05:01-DQB1*02:01 in homozygosis) and *trans* conformations (DQA1*02:01-DQB1*02:02 with DQA1*05:05-DQB1*03:01 in heterozygosis), associated with the highest probability (20%) of developing CD. The group 2 (intermediate risk) included those infants carrying the DQ2 haplotype in *cis* conformation along with any other haplotype, as well as infants carrying the DQ8 haplotype (DQA1*03:01 DQB1*03:02) in homozygosis. This genotype is associated with a 7% probability of developing CD. The group 3 (low risk) included those infants with other common genotypes not associated with CD.

Ten infants were diagnosed with CD at the age shown in Table 1. The disease was diagnosed according to the 1990 criteria of the European Society of Pedriatric Gastroenterology, Hepatology and Nutrition (ESPGHAN) based on symptoms, positive anti-tissue transglutaminase (tTG) antibodies, quantified using a tTG-IgA ELISA kit (Pharmacia Diagnostics, GmbH), and the histologic analysis of duodenal biopsy demonstrating villous atrophy compatible with Marsh 3 classification. From the same cohort, a subset of best-matched control children (*n* = 10) regarding the type of delivery, HLA-DQ genotype, and type of feeding were selected for comparative purposes. In both groups, exclusion criteria included the onset of other disorders related to immune dysregulation such as type-1 diabetes, allergies, and IgA deficiency.

### Fecal sample processing

Fecal sample processing was done as described previously [24]. Briefly, fecal samples were diluted 1:10 (*w*/*v*) with PBS (130 mM NaCl and 10 mM Na_2_HPO_4_, pH 7.4) and homogenized. Aliquots submitted to low-spin centrifugation (600*g*, 2 min, at 4 °C) were used for DNA extraction (FastDNA™ SPIN kit for Soil, MP Biomedicals, Santa Ana, CA, USA). Aliquots submitted to high-speed centrifugation (15,000*g*, 10 min, at 4 °C) were used for immune marker quantification.

### Immune marker quantification

Calprotectin was quantified by diluting fecal samples in the calprotectin sample solution and using the ELISA Quantitative Fecal Calprotectin kit (DIAsource, Louvain-la-Neuve, Belgium). Secretory IgA (sIgA) and cytokines (tumor necrosis factor (TNF)-α, interferon (IFN)-γ, interleukin (IL)-1β and IL-6) were quantified using ELISA commercial kits (Bethyl, Montgomery, TX, USA) and Max Deluxe set (Biolegend, San Diego, CA, USA) respectively. The threshold sensitivity level for sIgA was 7.8 ng/mL; for TNF-α, IFN-γ, and IL-6 was 7.8 pg/mL; and for IL-1β was 2.0 pg/mL.

### Sequencing of 16S rRNA gene amplicons

The V1-V2 variable region of the bacterial 16S rRNA gene was amplified from the fecal DNA by PCR as described previously [25] and sequenced using the Illumina MiSeq platform (2 × 250 bp reads).

### Sequence analysis

The 16S rRNA gene sequence data were processed using the Mothur software package [26] as described previously [25]. After filtering to remove poor quality sequences and chimeras, each sample was rarefied to 2698 reads to ensure equal sequencing depth for all subsequent comparisons. Good’s coverage estimates at this sequencing depth averaged 99.6% (minimum of 99.2%). Clusters were analyzed using the Jaccard calculator, based on the presence/absence of operational taxonomic units (OTUs), and the Bray-Curtis calculator, based upon the presence/absence of OTUs and also on the relative abundance of each OTU. Diversity indexes (Shannon and inverse Simpson) were calculated using Mothur. OTUs were classified taxonomically by matching against the reference RDP database provided at the Mothur website (https://www.mothur.org/wiki/RDP_reference_files#Version_10). Classification of selected OTUs was verified using MegaBLAST against the NCBI database.

### Statistical analyses

All data analyzed in this study are shown in Additional files 1 and 2: Tables S1 and S2. Data distribution was assessed with the Shapiro-Wilk *W* test (SPSS software V19). Of the demographic data, the categorical variables were analyzed using the chi-square test (GradPad, 6.0) and the continuous variables (size, weight, and weeks of gestation at birth) using a *t* test (SPSS software V19). Comparisons of data of immune parameters between groups were done using the *t* test for non-paired samples and within groups over time were done using the *t* test for paired samples (SPSS software V19). Compositional comparisons at the OTU, genus, family, and phylum levels were carried out using metastats [27] and LEfSe [28]. False discovery rate was used for *p* value correction upon multiple comparisons using the Benjamini-Hochberg method [29]. With metastats, the relative abundance of each OTU was compared across the two populations (CD and control) by computing a two-sample *t* statistic. The null distribution of *t* was estimated non-parametrically using a permutation test [27]. For diversity measures, the Mann Whitney *U* test was used for comparisons between infant groups and the Wilcoxon signed-rank test for comparisons within groups at different ages using R v3.12. Correlations between the microbiota composition and immunological parameters were established using the Spearman coefficient (GradPad, 6.0). Statistically significant differences were established at *p* < 0.05.

## Results

### Demographic data, genetics, and early feeding pattern of the infants

A nested case-control study was conducted to investigate the role of the evolution of the infant gut microbiota in CD onset in a subset of subjects selected from a larger prospective cohort study [23]. The Table 1 shows the baseline characteristics of the infants included in the study. These were similarly distributed between the two groups (*p* < 0.05), except for height at birth, which was higher in the controls than in the CD group (50.2 versus 48.8 cm, *p* < 0.05). Regarding the type of feeding, at 4 months of age, 3 subjects were fed with formula and 7 were breast-fed in both the control and CD group. According to pediatricians’ recommendations, all infants started the transition from milk feeding to the first solid foods between 4 and 6 months of age. The introduction of gluten was initiated from 6 months of age onwards in all cases. From birth to 4 months of age, no infections or antibiotic intake were registered. Up to 6 months, a total of 6 infants suffered from infections during the time frame of our study and were treated with antibiotics. Infant CD2 suffered from pharyngitis; CD3 suffered from an upper respiratory tract infection; CD5 suffered from otitis; CD6 and CD8 suffered from an upper respiratory tract infection and also from otitis, and control C5 suffered from bronchitis. No significant differences (*p* = 0.143) were detected between groups in the intake of antibiotics.

The diagnosis of CD for most cases occurred between the ages of 16–40 months (mean 34.2 months, SE 5.9), with the exception of one case (CD9), who was diagnosed at 82 months of age. At the time of diagnosis, all CD children had elevated levels of tTG antibodies and the following symptomatology: four cases presented diarrhea and abdominal distension, one constipation and asthenia, and five had no symptoms.

### Early evolution of gut microbiota in relation to CD development

**Fig. 1:**
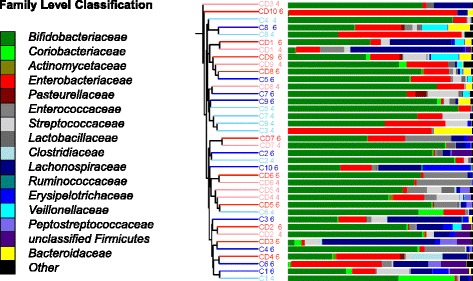
Jaccard dendrogram colored by disease status, with controls at 4 months (dark blue), controls at 6 months (light blue), and children who developed CD (CD group) at 4 months (pink) and at 6 months (red)

Overall cluster analysis of the 16S rRNA gene sequence data, using either the Jaccard or the Bray-Curtis calculators, showed there was little distinct clustering of the early microbiota profiles by disease status later in life (Fig. 1). However, the parsimony test as implemented in Mothur software [26] suggested that there was a borderline significant difference in bacterial community membership (Jaccard) (*p* = 0.045), but not in the community structure (Bray-Curtis) at 4 months of age between the two groups of infants. This significance disappeared by 6 months (*p* = 0.640).

**Fig. 2:**
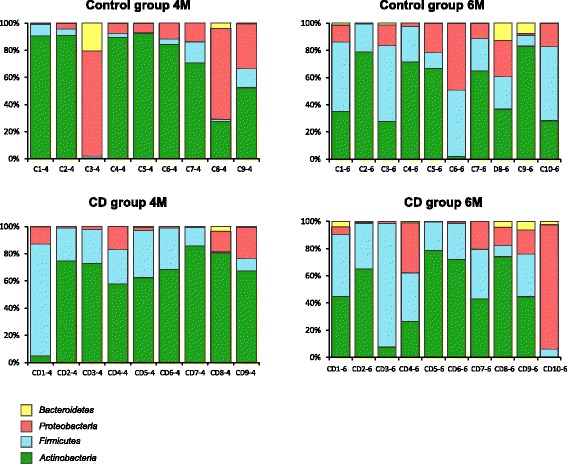
Proportion of the different phyla detected at 4 months (4M) and 6 months (6M) of age in children who developed celiac disease (CD group, “CD”) and who remained healthy (control group, “C”). The phyla are represented with different colors indicating that they belong to *Bacteroidetes* (yellow), *Proteobacteria* (red), *Firmicutes* (blue), and *Actinobacteria* (green)

Compositional analysis revealed the presence of eight phyla across the whole dataset, but only four of them were above the mean value of 1% of the total number of reads. The relative abundance of reads corresponding to *Bacteroidetes*, *Proteobacteria*, *Firmicutes*, and *Actinobacteria* in the CD and control groups at 4 and 6 months are represented in Fig. 2. At 4 months of age, children that later developed CD harbored a significantly higher proportion (*p =* 0.036) of *Firmicutes* compared to those that remained healthy (control group). This generally occurred alongside proportional reductions in *Proteobacteria* and *Bacteroidetes*, although the differences in these two phyla were not significant. At 6 months, no differences at the phylum level were observed between the two groups of children (Fig. 2).

We also evaluated the possible influence of covariates on gut microbiota composition, which could bias the results of comparisons between infants that remained healthy and those who developed CD later in life. No differences (*p* > 0.05) depending on the *HLA-DQ* genotype (high risk versus intermediate risk), the type of milk feeding (breast milk versus formula together or not with complementary feeding), and the effect of antibiotic intake in the samples collected at 6 months of age (positive intake versus negative intake) were detected with metastats after correcting for multiple comparisons.

**Fig. 3:**
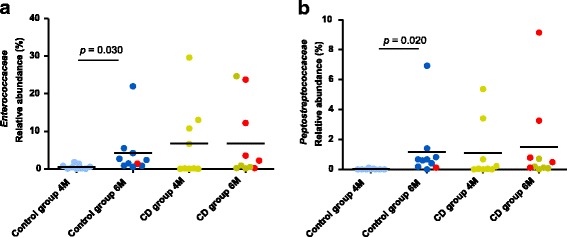
**a**, **b** Significantly different families detected at 4 months (4M) and 6 months (6M) of age in children who developed celiac disease (CD group) and who remained healthy (control group). Statistically significant differences were established at *p* ˂ 0.05. Red dots correspond to the infants that were exposed to antibiotics from 4 to 6 months of age

The early microbiota of CD children and the control group showed a different developmental process regarding the relative abundance of *Firmicutes*. In control children, *Firmicutes* proportions showed a statistically significant increase from 4 to 6 months of age (*p* = 0.011), while in the CD children, differences were not detected over time (Fig. 2). At both 4 and 6 months, no differences were observed between groups of children in the most abundant bacterial families and genera. When considering the gut microbiota development over time within groups, children that remained healthy showed proportional increases in *Enterococcaceae* (*p* = 0.030) (Fig. 3a) and in *Peptostreptococcaceae* (*p* = 0.020) (Fig. 3b) from 4 to 6 months of age, whereas no differences were found in the CD group over time.

We also used the LEfSe software program to detect putative bacterial biomarker species that could predict later development of CD [28]. At the level of the individual OTUs (a total of 374—see Additional file 1: Table S1), LEfSe analyses indicated that increased relative abundance of *Bifidobacterium longum* was associated with the control group (Linear Discriminant Analysis (LDA) = 5.004, *p* = 0.021), while that of *Bifidobacterium breve* (LDA = 4.846, *p* = 0.032) and *Enterococcus* spp. (*Enterococcus gallinarum/casseliflavus/faecium*, LDA = 4.507, *p* = 0.018) were associated with the CD cases. The presence of *Clostridium innocuum* (LDA = 4.005, *p* = 0.047), *Veillonella dispar/parvula* LDA = 3.867, *p* = 0.009), and *Intestinibacter bartlettii* (LDA = 3.415, *p* = 0.003) were associated with the age of 6 months.

### Richness and diversity analyses

**Fig. 4:**
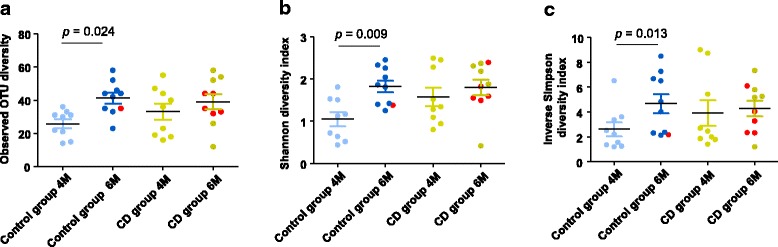
Number of observed OTUs (**a**), Shannon (**b**), and the inverse Simpson (**c**) diversity indexes detected at 4 months (4M) and 6 months (6M) of age in children who developed celiac disease (CD group) and who remained healthy (control group). Values are expressed as means and standard error of means. Statistically significant differences were established at *p* < 0.05. Red dots correspond to the infants that were exposed to antibiotics from 4 to 6 months of age

Microbial diversity was analyzed in both groups at 4 and 6 months (Fig. 4). The control group that remained healthy showed a statistically significant increase in both richness and diversity over the study period, as measured by the number of observed OTUs (*p* = 0.024) (Fig. 4a), the Shannon diversity index (*p* = 0.009) (Fig. 4b), and the inverse Simpson diversity index (*p* = 0.013) (Fig. 4c). In contrast, there was no significant increase in the microbial diversity over the study period in the group of children who developed CD later in life (*p* = 0.155, *p* = 0.193, and *p* = 0.407, for comparisons of number of OTUs, Shannon, and inverse Simpson indexes, respectively).

### Quantification of immune markers

**Fig. 5:**
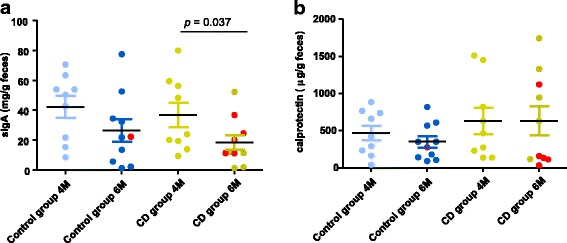
Secretory IgA (sIgA) (**a**) and calprotectin (**b**) in feces collected at 4 months (4M) and 6 months (6M) of age from children who developed celiac disease (CD group) and who remained healthy (control group). Values are expressed as means and standard error of means. Statistically significant differences were established at *p* < 0.05. Red dots correspond to the infants that were exposed to antibiotics from 4 to 6 months of age

Children who developed CD showed a significant reduction (*p* = 0.037) in fecal sIgA levels from 4 to 6 months of age, while the reductions did not reach statistical significance in children that remained healthy (Fig. 5a). No differences in the calprotectin levels were observed between groups at 4 months or 6 months or within groups over the study period (Fig. 5b). There was huge variability in calprotectin levels between individuals, which was more pronounced in the case of the CD children. In this group, the coefficient of variation (CV) was 85.0% at 4 months, and 103.2% at 6 months.

**Fig. 6:**
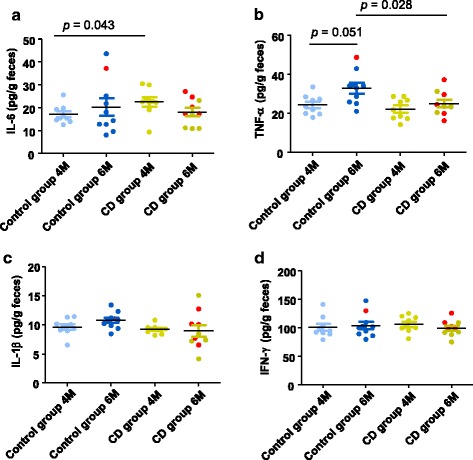
Cytokine levels, interleukin (IL)-6 (**a**), tumor necrosis factor (TNF)-α (**b**), IL-1β (**c**), and interferon (IFN)-γ (**d**) in feces collected at 4 months (4M) and 6 months (6M) of age from children who developed celiac disease (CD group) and who remained healthy (control group). Values are expressed as means and standard error of means. Statistically significant differences were established at *p* < 0.05. Red dots correspond to the infants that were exposed to antibiotics from 4 to 6 months of age

Regarding differences in cytokines, children who developed CD had higher levels of intestinal IL-6 (*p* = 0.043) than the control group at 4 months, but this difference disappeared at 6 months (Fig. 6a). The concentration of TNF-α tended to increase over time only in control children (*p* = 0.051; Fig. 6b), which might be linked to maturation of the gut microbiota and its increased diversity. Comparisons between groups also revealed that the control group showed higher TNF-α levels than the CD group (*p* = 0.028) at 6 months (Fig. 6b). No differences were observed in the IL-1β or IFN-γ levels between or within groups (Fig. 6c, d).

At 4 months, sIgA concentrations were positively correlated with *Bacteroidaceae* (*r* = 0.778, *p* = 0.017) and *Enterobacteriaceae* (*r* = 0.733, *p* = 0.031) and negatively with *Lachnospiraceae* (*r* = − 0.895, *p* = 0.002) and *Coriobacteriaceae* (*r* = − 0.712, *p* = 0.037) in the control group. Besides, a positive association was found between *Firmicutes* and calprotectin (*r* = 0.857, *p* = 0.024). At 6 months, the control group showed a positive correlation between *Bifidobacteriaceae* and TNF-α (*r* = 0.730, *p* = 0.020) and two negative ones between “unclassified *Lachnospiraceae*” and IFN-γ levels (*r* = − 0.644, *p* = 0.049) and *Bacteroidaceae* and IFN-γ levels (*r* = − 0.831, *p* = 0.005). This latter negative correlation was also found in the CD group at the same time point (*r* = − 0.806, *p* = 0.007).

## Discussion

This is the first study to identify features of the bacterial community assembly and intestinal immune milieu that precede CD onset and that may constitute predictive markers of disease risk and modifiable risk factors. Although there were not great differences in microbiota composition between the two infant groups (those who later developed CD or those who remained healthy), specific differences were identified in the early evolution of bacterial communities and of immune markers associated with subsequent CD development.

Our study shows a significant increase in microbiota diversity of control children between the ages of 4 and 6 months, which did not occur in the child group who went on to develop CD. Similarly, specific bacterial communities followed a different trajectory over time in the study groups, revealing differences in the phylum *Firmicutes*, and the families *Enterococcaceae* and *Peptostreptococcaceae* belonging to this phylum, whose proportions increased from 4 to 6 months only in those children who did not develop CD. Previous studies reported that the phylogenetic diversity of the microbiota in the first stages of life increases over time [30–32]. It was also estimated that the Gini-Simpson α-diversity index increases by 0.008 per day on average during the first month of life [33]. However, in the child group who went on to develop CD, diversity did not increase significantly over time, showing an uncommon developmental pattern (“premature maturation”) compared to the healthy group, since they started with a higher basal diversity level. Therefore, a progressive increase in microbial diversity seems to be a feature of the evolution of a healthy microbiota, which may increase resilience to CD. A previous prospective study with only one case developing CD at 24 months of age also reported reductions in bacterial richness before disease onset (6 to 10 months of age) [34]. However, the findings of the aforementioned study are of limited value since statistical analysis could not be performed with a single CD case. Furthermore, the results are incomparable since the cited study aimed to investigate how CD onset was affected by the time the gluten was introduced into the diet, introducing different variables from those considered in our study.

A number of observational and to a lesser extent intervention studies in adults have generally associated an increased bacterial diversity with a healthy phenotype [35–37]. The present study, however, suggests that a timely maturation of the gut microbiota towards one of higher complexity and diversity might also be required for proper development of immune tolerance in the early stages of life and that premature maturation and exposure to a complex microbiota may increase disease risk.

In our study, a control group of infants matching the disease group regarding the type of milk feeding, mode of delivery, and the HLA genotype was selected for comparative purposes. Therefore, the early increase in diversity observed in the infants who subsequently developed CD could be due to other uncontrolled environmental exposures that influence colonization waves of the infant’s gut (perinatal factors, hospital and home environment, number of siblings, etc.) or additional genetic factors that create permissive conditions to accommodate a more heterogeneous microbiota. Although the effect of host genetics on the microbiota is less known than the effects of environmental factors, associations between the microbiome and genes related to innate immunity that could control gut colonization (e.g., pattern recognition receptors) have been identified through genome-wide association studies [38].

Intestinal infections and their treatment with antibiotics have been associated with an increased risk of developing CD in some studies [13]. However, our study infants only suffered from otitis and respiratory tract infections during the study period and the antibiotic treatment was not significantly associated with CD onset. A possible impact of antibiotic intake on the microbiota of our infants could not be completely disregarded due to the relatively small size of the study. Nonetheless, the microbiota of those infants who later developed CD already presented a pattern of maturation significantly different to the control group at 4 months of age (before the intake of antibiotics), according to the differences in the bacterial community membership (Jaccard similarity index) that were attributed to increases in *Firmicutes*.

Taking into account the CD genetic risk classification of the infants included in the study, we did not find significant association with the intestinal microbiota composition either. These results are in agreement with our previous studies reporting no major differences in microbiota composition as a function of genotype when comparing risk groups 1 and 2 by qPCR [23]. Notwithstanding, we did find differences when comparing the high-risk groups 1 and 2 with the low-genetic risk group 3 [23] and also when comparing the highest risk infants (group 1) with low-risk infants (group 3) [24].

In our previous studies on CD, we found that the high-risk genotype for developing CD was associated with reduced numbers of *Bifidobacterium* (*Actinobacteria* phylum), specifically of the species *B*. *longum* [23] compared to the low-risk genotype. Accordingly, at that time, we hypothesized that microbiota differences associated with the HLA-DQ2/8 genotype could constitute an additional risk factor for CD onset. The present results further support the hypothesis that a reduced abundance of *B*. *longum*, dependent on both genetic and environmental factors, may constitute a risk factor for CD development and constitute an early predictive biomarker of CD.

Our present study also suggests that early proportions of *Firmicutes* and members of the *Actinobacteria* phylum (*B*. *longum*) in infants at familial risk of developing CD are influenced not only by the HLA-DQ2/8 genotype [23], but also possibly by other genetic and environmental factors. However, considering the complexity of the *Firmicutes* phylum, larger cohort sizes and in-depth taxonomic studies will be needed to understand the potential biological role played by specific components of this phylum and their role in CD onset in response to other environmental factors.

Interestingly, we also observed a faster reduction in sIgA fecal levels in the children who went on to develop CD over time compared to healthy ones. sIgA is generally considered to act as a first line of defense in the intestine by interacting with intestinal antigens and microbes, preventing their penetration into the lamina propria, and therefore contributing to immunological homeostasis [39]. sIgA is also thought to play a role in mucosal sensing and control of commensal bacteria colonizing the gut [40]. Besides, sIgA influences the development of dendritic cells with a tolerogenic phenotype, which play a key role in maintaining intestinal homeostasis [41, 42]. Thus, it can be hypothesized that a premature reduction of sIgA levels in the group of children that developed CD could be related to shifts in bacterial community development which impact the maturation of the mucosal immune functions, possibly increasing the risk of developing autoimmune dysfunctions. Moreover, the fact that CD frequently occurs in subjects with selective IgA deficiency has been interpreted as an indicator that there is a reduction in the infant’s mucosal protection [6].

The differential profiles of fecal cytokines over time in the two groups of infants may also reflect differences in gut microbiota maturation. Early increased levels of IL-6 could be related to premature maturation of the microbiota of infants who finally developed CD, while increased levels of TNF-α at 6 months could reflect the host response to a more diverse microbiota acquired at this age by infants who remained healthy, and to its specific components such as *Bifidobacterium*, which showed a positive correlation with TNF-α.

The strength of this report lies in the exhaustive prospective follow-up of the study cohort, where bias that usually affects retrospective studies has been excluded. Furthermore, a nested case-control study has integrated genotypic, dietary, immune, and microbiota data from biopsy-verified CD cases for the first time. The main limitation is the relatively small population size of the prospective study, taking into account the low prevalence of CD in this at-risk population (~ 10%). This limits the statistical power to investigate the effects of different covariates, including the possible role of antibiotics, which would have helped to reach more robust conclusions.

## Conclusions

Overall, we demonstrate shifts in the early trajectory of the gut microbiota along with changes in immune markers in infants who later develop CD. These alterations are indicative of deviations of the normal microbiota maturation process, which precede CD development. Further studies that build on these observations are warranted to progress in the understanding of the specificities of these deviations and the mechanisms by which they might increase disease risk in response to environmental triggers.

## Additional files

Additional file 1: **Table S1.** Full list of OTUs and taxonomic classifications. (XLSX 87 kb)

Additional file 2: **Table S2.** Ecological descriptors and immune parameters. (XLSX 12 kb)

## Abbreviations

APCs: Antigen-presenting cells
CD: Celiac disease
ESPGHAN: European Society of Pedriatric Gastroenterology, Hepatology and Nutrition
GFD: Gluten-free diet
HLA: Human leukocyte antigen
IFN: Interferon
IL: Interleukin
OTUs: Operational taxonomic units
sIgA: Secretory IgA
TNF: Tumor necrosis factor
tTG: Tissue transglutaminase
